# Cognitive and structural predictors of novel task learning, and contextual predictors of time series of daily task performance during the learning period

**DOI:** 10.3389/fnagi.2022.936528

**Published:** 2022-09-23

**Authors:** Evan T. Smith, Paulina Skolasinska, Shuo Qin, Andrew Sun, Paul Fishwick, Denise C. Park, Chandramallika Basak

**Affiliations:** ^1^Center for Vital Longevity, University of Texas at Dallas, Dallas, TX, United States; ^2^Department of Psychology, University of Texas at Dallas, Richardson, TX, United States; ^3^School of Arts and Technology, University of Texas at Dallas, Richardson, TX, United States

**Keywords:** game learning, cognitive training, time-series analysis, aging, gray matter volume, game intervention design

## Abstract

Investigation into methods of addressing cognitive loss exhibited later in life is of paramount importance to the field of cognitive aging. The field continues to make significant strides in designing efficacious cognitive interventions to mitigate cognitive decline, and the very act of learning a demanding task has been implicated as a potential mechanism of augmenting cognition in both the field of cognitive intervention and studies of cognitive reserve. The present study examines individual-level predictors of complex skill learning and day-to-day performance on a gamified working memory updating task, the BirdWatch Game, intended for use as a cognitive intervention tool in older adults. A measure of verbal episodic memory and the volume of a brain region involved in verbal working memory and cognitive control (the left inferior frontal gyrus) were identified as predictors of learning rates on the BirdWatch Game. These two neuro-cognitive measures were more predictive of learning when considered in conjunction than when considered separately, indicating a complementary effect. Additionally, auto-regressive time series forecasting analyses were able to identify meaningful daily predictors (that is, mood, stress, busyness, and hours of sleep) of performance-over-time on the BirdWatch Game in 50% of cases, with the specific pattern of contextual influences on performance being highly idiosyncratic between participants. These results highlight the specific contribution of language processing and cognitive control abilities to the learning of the novel task examined in this study, as well as the variability of subject-level influences on task performance during task learning.

## Introduction

Investigation of factors that influence successful learning has a long history in the psychological sciences. Aside from obvious importance to the fields of learning and skill development, the question of what factors influence individual learning rates is also of central importance to the field of cognitive training in normal aging. Several investigations of cognitive training have found that learning outcomes during the training period directly relate to training outcomes in terms of transfer to unrelated or “far” cognitive measures (Basak et al., 2008; Bürki et al., 2014; Basak and O'Connell, 2016). Based on their findings, Bürki et al. (2014) concluded that an understanding of the individual difference factors that influence the learning of the training task is a critical step in the development of efficacious cognitive intervention, and other researchers have expressed a similar position (Taatgen, 2013; Gathercole et al., 2019). Past research in this domain has revealed several cognitive and brain structure factors which appear to predict success in novel complex task learning in older adults (Erickson et al., 2010; Basak et al., 2011; Ray et al., 2017; Smith et al., 2020).

Both cognitive and structural predictors of learning novel, computerized tasks have been identified by past research. Ray et al. (2017) reported that measures of working memory were predictive of learning rates of two novel video games in a lifespan sample. This finding was later replicated by Smith et al. (2020). In addition to working memory, Ray et al. (2017) found a measure of perceptual discrimination (cued discrimination task; Posner, 1980) to be predictive of learning for the strategy game that relied more on working memory and cognitive control than the action game. In terms of structural predictors, in younger adults, Erickson et al. (2010) demonstrated that individual differences in the gray matter volume (GMV) of the striatum predicted learning outcomes on a lab-developed game-like computer task designed to stress working memory, cognitive control, and response time. In older adults, Basak et al. (2011) identified a number of predominantly left fronto-parietal gray matter regions (including left medial frontal gyrus, left dorsolateral prefrontal cortex, anterior cingulate cortex, and left postcentral gyrus) and cerebellum, whose volumes predicted learning of a commercial real-time strategy video game, which had shown transfer to laboratory-based measures of cognitive control, working memory, and reasoning (Basak et al., 2008). White matter correlates of novel computer task learning have also been identified: Ray et al. (2017) identified two discreet white matter microstructures (left cingulum-hippocampus and right fornix-stria terminalis), the integrity of which predicted the learning rate on two commercial video games. Importantly, left cingulum-hippocampus integrity predicted learning in the strategy game in both young and old adults. In sum, left fronto-parietal gray matter volumes and structural connectivity between the hippocampus and frontal cortex have been predictive of novel strategy game learning in older adults.

Another factor that may strongly contribute to individual differences in task learning, especially in older adults, is cognitive reserve. Cognitive reserve is known to be predictive of performance on episodic and working memory tasks, executive function, speed of processing, and general cognition (Opdebeeck et al., 2016). Considering that all of these factors are likely invoked in the learning of a complex, novel task, such as those used in cognitive training interventions (Gathercole et al., 2019), and the known relationship between cognitive reserve and retained cognitive function in later life (Park et al., 2014; Bak et al., 2016; Ward et al., 2020), an investigation of how cognitive reserve interacts with novel task learning is similarly warranted.

As this body of work demonstrates, the field is continuously making strides in identifying individual difference factors that influence the learning of novel tasks. However, if our stated goal is to apply this knowledge to develop efficacious cognitive interventions for at-risk groups, particularly the elderly, the above-summarized research exhibits some limitations. First, most of the studies cited above used a young adult (Erickson et al., 2010) or lifespan sample (Ray et al., 2017; Smith et al., 2020), which limits the conclusions we can draw with regard to our target population, that is, older adults aged 65 years and above. Second, all but one of the above-cited studies (Basak et al., 2011 being the exception) utilized short-term learning periods of 2.5 h or less, which therefore limits any conclusions we can draw from this research to this early period of task learning. As most reported cognitive interventions in older adults are of a substantially greater length (for a meta-analysis, see Basak et al., 2020), an examination of how such predictors affect learning at a later training phase is warranted. Third, the act of task learning requires consistent invocation of episodic memory, working memory, and cognitive control (Taatgen, 2013), and these capacities are susceptible to a wide range of cognitive and psychosocial contextual factors (Stawski et al., 2011). Considering this, it is likely that such factors have a downstream influence on the task learning process itself, which may contribute to the large individual differences in patterns of task learning that have been observed (Bürki et al., 2014), but examinations of such contextual effects on performance during training tasks are lacking.

Based on the findings and limitations of the above-summarized research, the present study was designed to further examine cognitive and structural correlates of learning on a working memory training task, as well as daily contextual factors which may influence training task performance during the training period. Reasoning and episodic memory were selected as cognitive predictors in order to expand on the past research which has already established working memory ability as a correlate of task learning (Ray et al., 2017; Smith et al., 2020). To evaluate the cognitive and structural correlates and daily contextual factors of learning on a training task, we used data from a recently completed clinical trial in healthy aging (registered at ClinicalTrials.gov as NCT03988829), where variations of a PI-developed working memory training game (“BirdWatch Game”) were used as interventions. For the present study, we focused on the BirdWatch Game and baseline measures of hypothesized cognition and gray matter volume correlates of learning of that game. If episodic memory and reasoning interact with BirdWatch Game learning as working memory has been demonstrated to with other computerized task learning, we would expect participants with greater pre-training ability on those constructs to demonstrate more rapid learning of the BirdWatch Game, and potentially higher maximum attainment. Additionally, because the BirdWatch Game itself is a working memory updating training paradigm, initial performance on the BirdWatch Game can be interpreted as the baseline working memory ability (both capacity and updating) of participants in this study. By that conceptualization, we predict that individuals with greater initial performance on the BirdWatch Game will show more rapid learning of the task, in line with past research (Ray et al., 2017). We hypothesize that cognitive reserve will demonstrate a similar relationship to task learning as the other examined cognitive constructs, considering past research which has observed a correlation between cognitive reserve and initial task learning (Lojo-Seoane et al., 2020). Alternatively, lower cognitive construct/reserve measures prior to training may relate instead to greater improvement on the trained task due to lower initial performance, as similar results have been observed in some past cognitive training studies (López-Higes et al., 2018). We expect this alternate hypothesis to be supported by greater progress in late learning specifically, if indeed it is supported, considering the past evidence that relatively lower cognitive ability/reserve results in slow initial learning (Ray et al., 2017; Lojo-Seoane et al., 2020).

A recent meta-analysis on cognitive interventions across both healthy aging and older adults with mild cognitive impairments (Basak et al., 2020), which included 214 cognitive training studies, found that the immediate cognitive gains in the cognitive training group is significantly more than the control group (net gain effect size = 0.28, *p* < 0.001). Importantly, the most effective intervention that resulted in the largest effects of near and far transfer trained either executive functions or working memory. The PI and her team designed a computerized cognitive training intervention, the BirdWatch Game, based on the Theory of Working Memory Adaptability (Basak and O'Connell, 2016), which predicts that high cognitive control demands from unpredictable probe-cues during working memory updating engender greater far transfer than predictable probe-cues in healthy aging. However, Basak and O'Connell had used well-learned verbal stimuli (digits), and the training was not adaptive or gamified to ensure engagement. The BirdWatch Game features qualities found to be effective in past cognitive training, including adaptive scaling difficulty (Boot et al., 2010; Payne et al., 2011; Brehmer et al., 2012; Cuenen et al., 2016) and computer-based gamification with novel stimuli that induce greater engagement and show transfer in older adults (Lampit et al., 2014; for meta-analyses, see Basak et al., 2020).

Considering that the BirdWatch Game is a working memory updating task, we hypothesize that the gray matter volumes of regions known to be related to working memory and cognitive control (e.g., frontal gyri, anterior cingulate cortex, premotor cortex, etc.) will positively predict its learning. The volumes of areas known to be related to learning in general (i.e., hippocampus and striatum) are likely to demonstrate a similar pattern. Additionally, considering the length of the training period utilized in this study, this study may reveal a differential relation between some of these examined volumes and early vs. late stages of learning. Specifically, the volume of the hippocampus may selectively relate to initial learning of the BirdWatch Game, considering its critical role in declarative learning (Burgess et al., 2002; Lim et al., 2020), and the theoretical contribution of episodic memory function to the cognition-dependent and strategy-dependent first and second stage of procedural learning (Ackerman, 1988; Beaunieux et al., 2006). Conversely, the volume of the striatum may selectively relate to later learning of the BirdWatch Game considering that region's contribution to procedural/automatized learning which occurs at later stages (Saint-Cyr and Taylor, 1992; Simonyan, 2019).

In terms of day-to-day predictors of task performance, contextual factors of sleep duration, stress, busyness, and physical and emotional wellbeing were examined as determinates of day-to-day performance on the BirdWatch Game learning. Sleep quality and duration are positively related to multiple cognitive abilities (Holanda Júnior and de Almondes, 2016; Lo et al., 2016; Rana et al., 2018; Zavecz et al., 2020), but stress negatively impacts working memory and cognitive control (Shields et al., 2016; Plieger and Reuter, 2020). Subjective wellbeing is also a positive correlate of working memory and cognitive control (Luerssen and Ayduk, 2017; Ihle et al., 2021). A secondary goal of this study was to examine how these contextual factors contribute to day-to-day performance on the training task. These measures, assessed at the onset of each training session, are hypothesized to predict overall performance during that session. Specifically, we hypothesize that stress and hours of sleep will have a strong aggregate effect if high stress or a few hours of sleep recur over several sessions, whereas wellbeing will relate positively to training performance. Additionally, considering past evidence (Festini et al., 2016), busyness may also relate positively to training performance.

## Methods

### Participants

A total of 55 older adults participated in a randomized clinical trial (RCT) contrasting different computerized cognitive training methodologies in healthy older adults (Basak, NCT03988829), from which the present study drew data. Of the 43 participants randomized to the BirdWatch Game—Unity (BWGU) training, 37 participants (*M*_*age*_ = 71.57, *SD*_*age*_ = 4.23, 54% female) completed both baseline cognitive assessments and BWGU training period sufficient to be included in the present study. The remaining participants either explicitly ceased involvement in the study due to the outbreak of the COVID-19 pandemic in early 2020 or ceased responding to scheduling requests during the period of the pandemic.

Of the 37 participants included in this analysis, seven participants were unable to complete the structural MRI scans due to the periodic unavailability of MRI scanners due to the COVID-19 pandemic, as outlined above, resulting in a sample size of 30 participants (M_age_ = 71.17, SD_age_ = 4.21, 57% female) who contributed cognitive, MRI, and training data sufficient to be included in all the analyses presented below. Additionally, the difficulties of collecting data *via* in-person testing during the 2020–2021 COVID-19 pandemic resulted in a higher than expected number of participants with missing data (*n* = 7). Five participants were unable to contribute CRIq data due to technical difficulties arising from remote data collection during the period of the pandemic. Analyses presented in the following sections for which some participants were excluded due to missing data are explicitly noted.

### Development of the BirdWatch game cognitive training program

At the core of this intervention program, titled the BirdWatch Game—Unity (BWGU), is the n-match paradigm, a modified n-back task in which participants must maintain and unpredictably update a number of items in their working memory simultaneously (Oberauer, 2006; Basak and Verhaeghen, 2011; Basak and O'Connell, 2016; O'Connell and Basak, 2018). In a typical n-back paradigm, participants are presented with a continuous sequence of individual stimuli and asked to compare the currently presented stimuli with the stimuli presented n items ago (Owen et al., 2005). Performing this task successfully requires participants to maintain the past *n* presented items within their working memory, continuously updating this information as new stimuli are presented (Jaeggi et al., 2010), and manipulating n in this paradigm thereby allows for the manipulation of participants' cognitive load.

The n-match paradigm (Basak and O'Connell, 2016) extends the traditional n-back paradigm by dynamically varying n during a single run of the task. This is accomplished by randomly presenting the stimuli in a set number of visuo-spatial contexts, and requiring participants to compare the currently displayed stimulus to the stimulus last displayed. For example, Basak and O'Connell (2016) utilized the numbers 1–9 presented in one to four different colors (the number of colors represented the n contexts of n-match task), and tasked participants with comparing the currently presented number with the most recent number presented in that same color. An earlier work by Basak and Verhaeghen (2011) utilized up to four different locations as contexts in an n-match paradigm to a similar effect. Due to the random presentation of context (color or location), participants are forced to actively maintain all *n* items within their working memory simultaneously and to unpredictably update this stored information, thereby increasing cognitive effort compared to a traditional n-back task where the n is fixed (Basak and Verhaeghen, 2011; Basak and O'Connell, 2016). The advantage of the n-match paradigm is that *n* can be dynamically varied by varying the sequence order of the context (e.g., Basak and O'Connell, 2016).

This intervention was based on the efficacy of executive function training in older adults of which working memory is an essential process (Basak et al., 2020), commonality of working memory issues as a subjective complaint in older adult populations (Newson and Kemps, 2006), and the theoretical efficacy of using working-memory-based training to address that complaint and contribute to general wellbeing (Luerssen and Ayduk, 2017). We elected to utilize the n-match training paradigm specifically as it has been shown to facilitate far transfer to measures of reasoning and episodic memory in older adults (Basak and O'Connell, 2016), and because the n-match tasks stressed working memory updating rather than just working memory span, which Miyake and Friedman (2012) identify as separate contributors to executive functioning.

**Figure 1:**
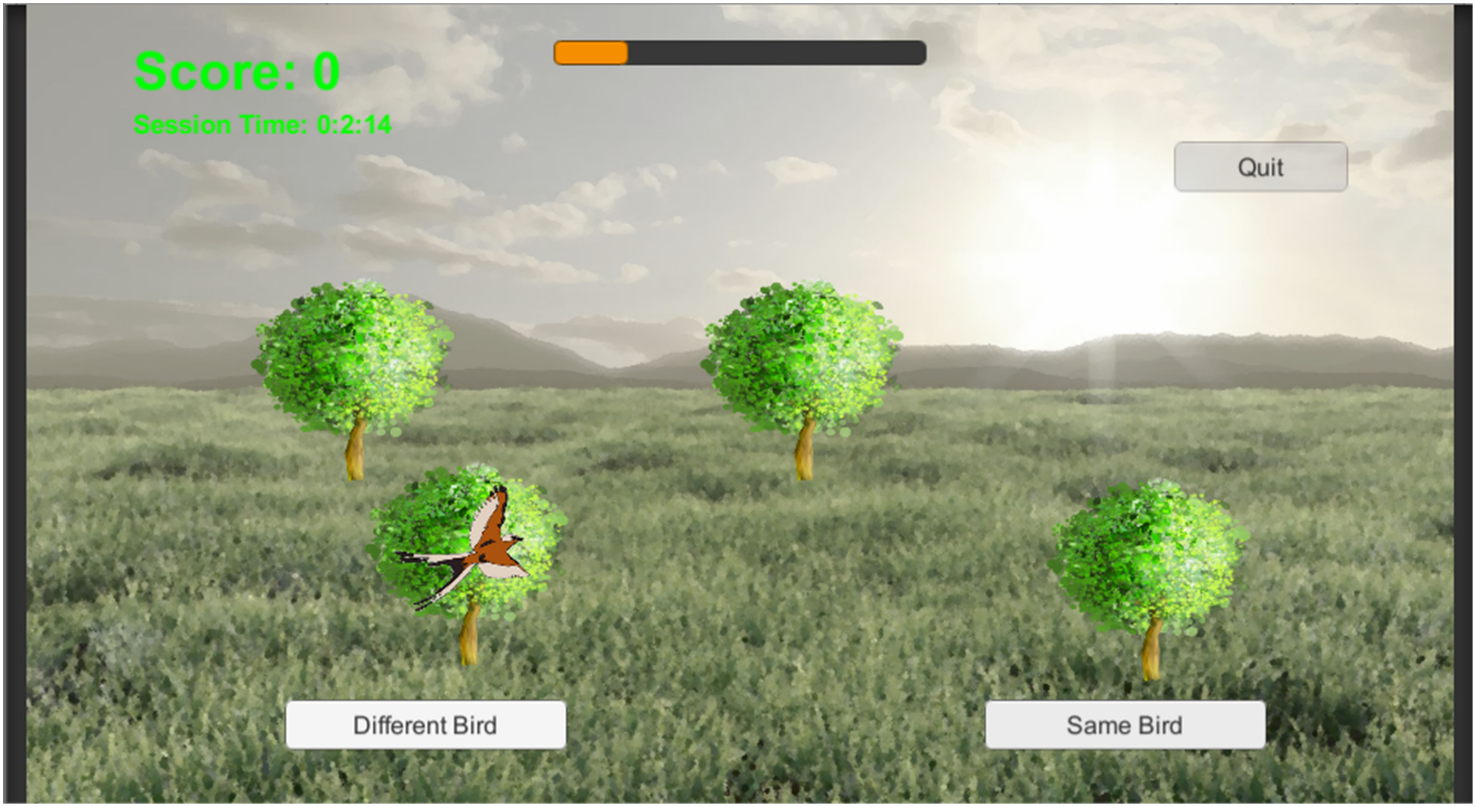
A single trial from BWGU, depicting a four-context trial.

The n-match paradigm described above was modified in several ways to produce the BWGU paradigm. First, to render the n-match paradigm more engaging, the paradigm was extensively gamified, i.e., modified to resemble a recreational video game. Simplified renderings of birds were used for individual stimuli, with trees in spatially distinct locations utilized as contexts (see Figure 1). Both bird stimuli and tree contexts are displayed on a rendering of an outdoor scene, selected to be both aesthetically pleasing and to reinforce the narrative that the BWGU training task is a “Bird Watching Game,” as implied by the title of the task.

Additionally, we added game-like player feedback to BWGU in the form of a score display and a “reward” system. The score was calculated as follows:


$$Score\;=\;100(Hit+CR)\;50(Miss+FA)\;+\;1000d^{\prime }(7-MaxRT)$$

In the above equation, *Hit* is the total number of hits from the previous block, *CR* is the total number of correct rejections from the previous block, *Miss* is the total number of misses from the previous block, *FA* is the total number of false alarms from the previous block, *d'* is the memory discriminability measure from the previous block, and *MaxRT* is the maximum allowed response time for the previous block (see below). While this scoring output is partially determined by performance metrics relevant to the goals of the present study, this score display was primarily implemented as an engagement tool that allowed participants to have a general sense of how their performance was progressing over time.

A “reward” system was implemented by the “unlocking” of new background images as participants met performance milestones, specifically whenever the performance threshold set by the program was increased (see below). This system was intended to somewhat reduce the monotony of performing the same task over multiple hours of training by periodically providing a different visual appearance over time, and to reinforce participant's success by tying this cosmetic change to performance milestones.

To further gamify this task, we implemented BWGU within the Unity game engine (Version 2018.4.2f1; 2018), a robust game development toolkit commonly used in independent game development. This allows BWGU to be deployed and run across multiple electronic platforms (i.e., Windows computers, Android and Apple phones, etc.) as if it were a recreational video game. As an added benefit, the Unity engine is sufficiently feature-rich and expandable to be comparable to data collection software more commonly used in cognitive science research (i.e., Eprime), which allowed for the collection of detailed performance metrics as described in the sections below.

**Figure 2:**
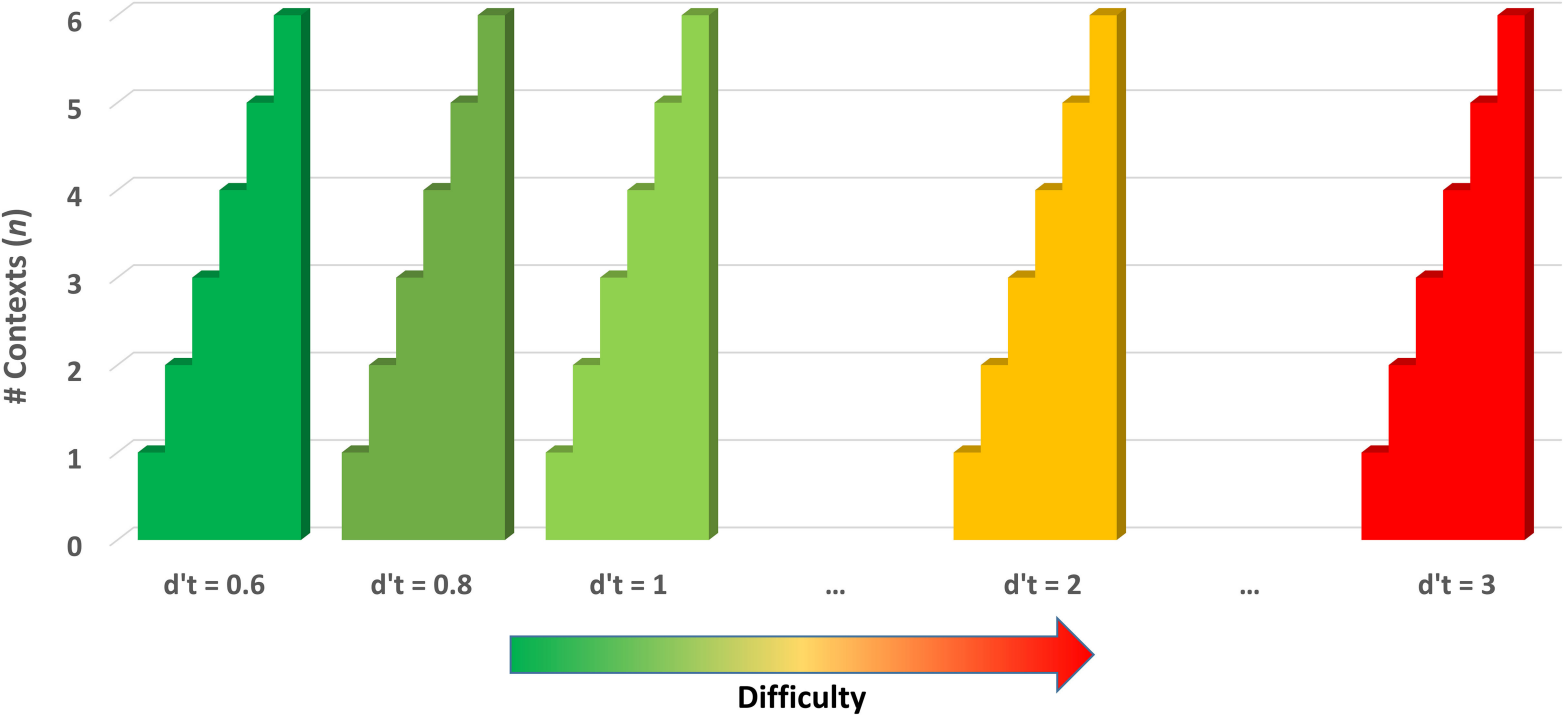
Depiction of overall difficulty progression by the number of contexts (*n*) and performance threshold (*d'*_*t*_) in the BWGU paradigm.

Several methods of adjusting the difficulty of the BWGU task based on the participant's real-time performance were implemented within the paradigm based on past research, which implicates individualized-adaptive training methodologies as efficacious (Mihalca et al., 2011; Payne et al., 2011; Brehmer et al., 2012; Cuenen et al., 2016). First, BWGU continuously adjusts the number of contexts, *n*, utilized for a given block of trials based on participant performance in the previous block. Discrimination accuracy (*d'*) was utilized as the measure of participant performance and was calculated as *Z*_*FA*_ –* Z*_*hit*_, where *FA* is the number of false alarms from the previous block, and *hit* is the number of correct identifications made in the last block. The 1/2N correction was applied to account for floor and ceiling effects (Macmillan and Creelman, 2005). The participant's *d'* for each block is compared to a performance threshold, *d'*_*t*_, and *n* is incremented by 1 for the next block if *d'* is greater or equal. BWGU scales up to six contexts. Should a participant perform above threshold, the performance threshold is increased, and the number of contexts is reduced to one. This increase in *d'*_*t*_ is associated with the “reward system” with each increase in *d'*_*t*_ “unlocking” a new background display. The performance threshold begins at 0.6, and increments by +0.2 for each participant's success on an *n* = 6 block, to a maximum of *d'*_*t*_ = 3. This system allows the BWGU paradigm to scale up the difficulty in response to an individual participant's performance up to 72 times (six contexts by 12 increases in threshold) over the course of training (see Figure 2).

Additionally, the response time window in which a participant is able to enter a response to the current stimuli also scales in two ways with participant performance. By default, participants have 5 s to respond to a new stimulus (i.e., *MaxRT* = 5 s). If an input is not detected in that time, that trial is marked as a “miss,” and the task progresses to the next trial. For each 10% of the total expected training time elapsed, *MaxRT* is decremented by 0.5 s to a minimum of 1 s. Conversely, for every three consecutive failures to pass the performance threshold at the end of a block of trials, *MaxRT* is incremented by 0.5 s, to a maximum of 6 s. In this way, time pressure is both increased and decreased in line with the participant's performance and progress through training.

### Implementation of BWGU in a multi-armed randomized controlled trial

The BWGU was utilized in two training arms of this RCT that contrasted various degrees of cognitive control over 20 h of training (Basak, NCT03988829). The two training arms of BWGU varied only in the sequence order of the context within a block, while all other features remained the same.

#### Recruitment

General inclusion criteria for the RCT were as follows: minimum age of 65 years, at least a 10th-grade education, learned English before the age of 5 years, and cognitively unimpaired (i.e., a Montreal Cognitive Assessment/MoCA score of 26 or greater; Nasreddine et al., 2005). Exclusion criteria included a history of cardiovascular disease other than treated hypertension, diabetes, psychiatric disorder, illness or trauma affecting the CNS, substance/alcohol abuse, and medication with anti-psychotics or hypnotics other than occasionally used at bedtime.

In addition to the above criteria, participants in the RCT were required to fulfill additional exclusion criteria in order to undergo the structural MRI portion of the study. Inclusion criteria for the MRI portion of the trial included right-handedness. Exclusion criteria for the MRI portion of the trial included metal medical implants, claustrophobia, and pregnancy. Initial recruitment for the RCT targeted only participants that fulfilled both the general and MRI inclusion/exclusion criteria outlined above. However, the onset of the COVID-19 pandemic in March of 2020 necessitated the expansion of the study to include participants who did not meet the criteria for MRI scans due to (a) high attrition of participants due to the pandemic, and (b) the necessity to conduct only remote cognitive testing between March 2020 and March 2021.

#### Training protocol and cognitive assessments at baseline

Participants in both BWGU arms were asked to train for 20 h over a period of 8 weeks on the BWGU paradigm. Participants were asked to train for 2.5 h each week, divided across two to three sessions. The training was performed at home using a 9.6' Android tablet computer provided to the participants, with the BWGU training program pre-installed on that device.

For the purpose of this longitudinal investigation, BWGU was configured to administer continuous blocks of 80 trials, with *n, d'*_*t*_, and *MaxRT* modulated between blocks as described in Section Development of the BirdWatch game cognitive training program. Between blocks, the BWGU training program pauses until the participant indicates they are ready to begin another block or chooses to exit the program. In the latter case, the current value of *n, d'*_*t*_, and *MaxRT*, as well as the total training time completed, are saved by the program for use the next time the participant activates the training program. An additional feedback mechanism, a “progress bar,” was added to the BWGU training program to aid participants in tracking their progress through training. This progress bar, which can be seen in the top center of Figure 1, fills relative to the participant's progression through the assigned 20 h of training, with the percentage of the bar filled reflecting the percentage of total training time elapsed.

Trial-wise performance data collected by the program includes participant accuracy, reaction time, and trial characteristics (switch trial and update trial). Block-wise performance data collected includes *Score, n, d'*, and *d'*_*t*_.

Cognitive reserve was assessed at baseline using the Cognitive Reserve Index Questionnaire (CRIq; Nucci et al., 2012). This self-report questionnaire assesses cognitive reserve as an aggregate effect of occupational, educational, and leisure activities over the lifetime, and has been demonstrated to both be independent of measures of general intelligence (Nucci et al., 2012) and reliable across a wide range of populations (Maiovis et al., 2016; Ozakbas et al., 2021).

Episodic memory measures administered at baseline and post-training included the Rey Auditory Verbal Learning Test (RAVLT; Bean, 2011) and the *Story Memory* sub-measure of the Mini-Mental State Examination (Folstein et al., 1975). The RAVLT is a word-list learning task of 15 that includes measures of simple learning, long-term memory (LTM) interference after distraction, LTM interference after delay, and multiple forms of LTM errors (source memory, semantic, and phonetic confusions). The Story Recall task is a modified word-list memory task in which the to-be-remembered items form a simple narrative separated into 34 distinct units. Participants are asked to read the story once, and then asked to recite it in as close to the original language as possible. An everyday test of memory was also administered, which included sub-measures of prospective memory, non-verbal recognition memory, and spatial-relational memory. However, the test proved infeasible to administer remotely, and as a result of this and the co-occurrence of the COVID-19 pandemic with data collection for this study, six participants were unable to contribute data for this everyday memory test. As a result of this, this test was dropped as an episodic memory measure in the analysis.

Reasoning measures administered at baseline and post-training included Visual Puzzles and Matrix Reasoning sub-measures of the Wechsler Adult Intelligence Scale, 4th edition (Drozdick et al., 2012). The Visual Puzzles test is a timed non-verbal reasoning test in which participants are presented with a series of puzzles of increasing difficulty. The Matrix Reasoning test is, similarly, a timed non-verbal reasoning test in which participants are presented with a series of incomplete visual patterns of increasing difficulty.

The current study used only the pre-training baseline assessments of the above-mentioned cognitive indices of far transfer (reasoning, episodic memory, and cognitive reserve).

#### MRI protocol

Baseline and post-training scanning protocols were conducted using a Siemens Magnetom Prisma scanner with a 32-channel head coil. High-resolution anatomical images were acquired using a transverse MPRAGE T1-weighted sequence with the following parameters: TR = 2,300 ms; TE = 2.26 ms; flip angle = 8°; acquisition matrix = 256 × 256; voxel size = 1 mm^3^; 208 slices.

Specific information regarding the additional neuroimaging scans and behavioral assessments can be found in the preregistration for the RCT (Basak, NCT03988829). Data from these additional scans were not examined, as the current study specifically examined brain volume predictors of BWGU learning.

#### Daily survey of subjective wellbeing and sleep

**Figure 3:**
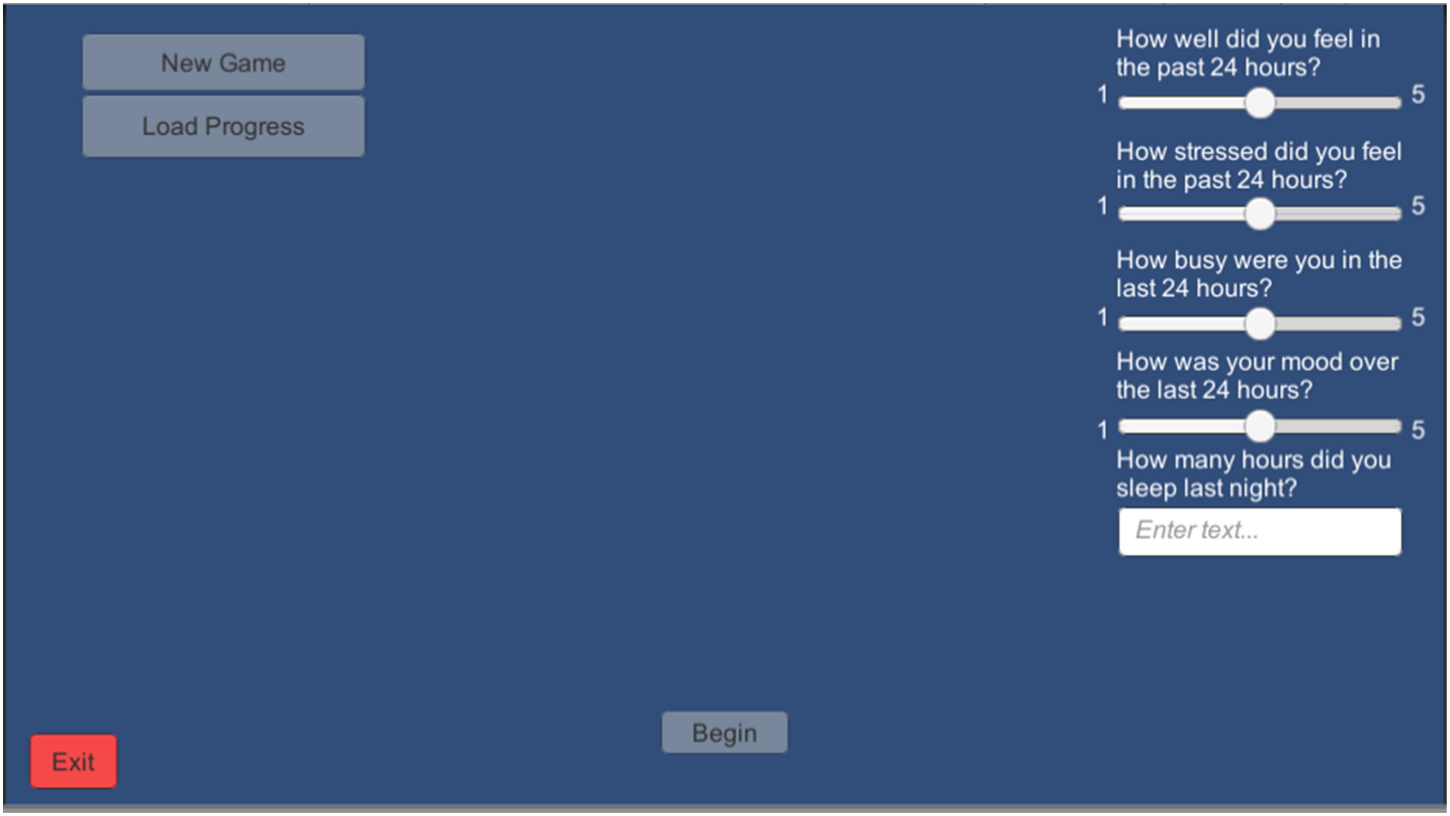
Screenshot of the Daily Survey Screen that appears just after the log-in screen in the BWGU.

To assess the impact of daily wellbeing on training performance-over-time, a short “daily survey” of subjective wellbeing and sleep measures was implemented in the BWGU training program. Participants were required to complete this survey each time they turn the program on, before their first block of training (see Figure 3).

The daily survey consists of a four-item Likert questionnaire on a 1–5 scale. Questions asked include (1) “How well did you feel in the past 24 h?” (2) “How stressed did you feel in the past 24 h?” (3) “How busy were you in the past 24 h?” and (4) “How was your mood in the last 24 h?” Questions 1 and 4 were presented on a scale from “1: very poor” to “5: very good,” and questions 2 and 3 were presented on a scale from “1: not at all” to “5: very.” Participant responses to questions 1 through 4 on this survey were taken as the Wellbeing, Stress, Busyness, and Mood variables, respectively. In addition to these Likert measures, participants were also asked to estimate their hours of sleep on the previous night, which was recorded as the Sleep variable.

### Analysis

#### Calculation of learning rates

The *Difficulty Level* of each block was assessed by counting the number of times that the BWGU had adaptively increased the demands of the task based on the participant's performance prior to the beginning of that block (see Section Participants). This calculation can be formally represented as follows:


$$Difficulty\;Level\;=\;6\frac{d_{t}^{\prime }\;-.6}{.2}+\;n$$

In the above equation, $d_{t}^{{}^{\prime }}$ represents the d-prime threshold of that block, and *n* represents the number of contexts for that block. Functionally, this results in the *Difficulty Level* for a block incrementing by +1 if either the number of contexts or the d' threshold has been updated since the previous training block. As the BWGU paradigm is designed to only adjust difficulty upward in response to player performance, we can correctly assume that any change in $d_{t}^{{}^{\prime }}$ or *n* to reflect an increase in difficulty, and therefore the total number of adjustments equates to the total difficulty of the training block. Assigning the first block of training the *Difficulty Level* of 1 results in a range of 1–72 for this variable (see Figure 2).

In order to differentiate performance on training blocks of the same difficulty level, the *Difficulty Level* per block was multiplied by that block's unscaled accuracy (hits + correct rejections, range 0–80, chance performance = 40), to produce a *Simple Score* for each block. This *Simple Score* variable was used to calculate learning rates for each participant, as described below.

**Figure 4:**
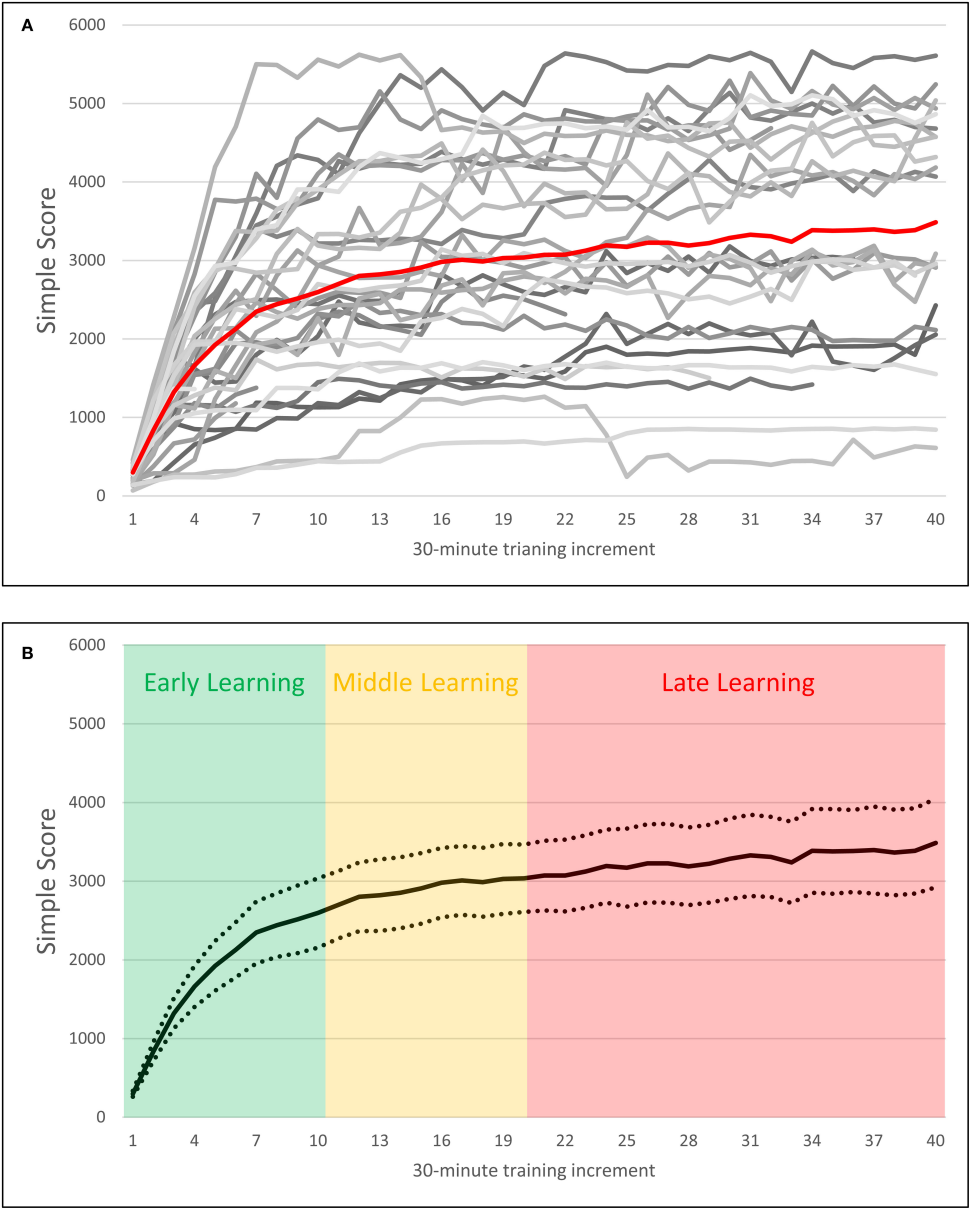
Plots of block-wise *Simple Score* by 30-min training increment over 20 h of training. **(A)** Depicts scores over time for individual participants represented in grayscale, with the average score over time plotted in red. **(B)** Depicts average scores over time with 95% confidence intervals, as well as demarcations of early, middle, and late training periods.

Past publications have used video game scores to calculate participant learning rates by fitting logarithmic curves to participants' scores over time, and taking the growth rate of that learning function as indicative of the rate of learning in older adults (Basak et al., 2011; Basak and O'Connell, 2016; Ray et al., 2017; Smith et al., 2020). Visual inspection of the *Simple Score* variable suggested that it followed a similar logarithmic pattern (see Figure 4), and so a similar method was employed in this study. The following logarithmic function was fit to each participant's *Simple Score* block-wise performance:


Y = b0 + (b1 ∗ ln(t))

In the above equation, *t* is the block of training (ordered sequentially, analog of training time/session), *Y* reflects the participant's *Simple Score* for a given *t, b*0 is the function's x-intercept, and *b*1 is the function's growth rate or slope. The growth rate of this function, as fitted to each participant's performance-over-time, was taken as that participant's *Overall Learning Rate*.

Compared to earlier studies that have used this method, the current study utilized a longer training intervention of 20 h (for an exception, see Basak et al., 2011, where training duration was 20 h). To account for this longer duration of the training, learning rates for early, middle, and late learning were calculated for each participant, corresponding to 1–5, 6–10, and 10–20 h of training, respectively, in addition to their *Overall Learning Rate*. The decision to define early, middle, and late learning in this way was based on a previous study where extensive practice on the n-back tasks in young adults stabilized after 5 h of training (Verhaeghen et al., 2004). As can be observed in Figure 4A, older participants also universally exhibited increasing performance across the first 5 h of training. Similarly, those participants who were able to reach asymptotic performance typically did so by the 10th h of training, as indicated by a relatively stable performance after 10 h of training. Based on these observations, hours 1–5 were designated as “early” training, and hours 10–20 as “late” training. The remaining period of hours 5–10 was designated as “middle” training, as the majority of participants appear to reach asymptotic performance within this range, but with specific achievement being time-variant.

An alternative approach to designating these training periods for the entire dataset would be to individually assign the early, middle, and late learning labels based on each individual subject's performance curve. However, we elected against this approach for two reasons. First, assigning learning periods across the whole group allows these data to be more readily comparable, a varying time period labeled as “early learning,” for example, would make interpretation of results related to that training period problematic. Second, defining those periods for the entire dataset rather than per participant reduces the potential for unconscious coder bias during the coder's division of the learning period for each individual.

Based on visual inspection of the learning data (see Figure 4B), logarithmic functions were fitted to participants' early learning period, with linear functions fitted to their middle and late learning periods. As with the *Overall Learning Rate*, the growth rate of the log function fitted to early learning data was considered as each participant's *Early Learning Rate*. Similarly, the slope of the linear functions fitted to participants' middle and late learning data was considered as each participant's *Middle Learning Rate* and *Late Learning Rate*, respectively. Due to variance in total training time, only *Early Learning* could be fitted for all participants. *Middle Learning* could be fitted for 32 of the 37 participants, and *Late Learning* could be fitted for 31 of the 37 participants. Information regarding variance in training time and compliance can be found in Results Section BWGU adherence and training outcomes.

#### Calculation of cognitive measures

As mentioned above, episodic memory measures administered before BWGU training included the Rey Auditory Verbal Learning Test (RAVLT; Bean, 2011) and the Story Recall sub-measure of the Mini-Mental State Examination (Folstein et al., 1975).

The RAVLT includes multiple outcomes of episodic memory, where target list A is learned across five trials (A1–A5), followed by incidental learning of non-target List B (B1), followed by a surprise recall of target list A (A6) after the interference from the non-target list, and 30-min delayed memory recall (A7) and recognition test for the target list (recognition A). Recognition of the target list also included source monitoring errors on the recognition trial (recognition B), semantic errors in the recognition trial (recognition SA), phonetic errors in the recognition trial (recognition PA), and compound source-semantic and source-phonetic errors on the recognition trial (recognition SB and PB). To simplify the outputs, we calculated several aggregate measures from the RAVLT's raw output. Trials A1 through A5 were summed to produce a measure of overall learning (*Learning Total*). The difference between trial A5 and A6 was taken as a measure of interference cost (*Interference Cost*). The difference between trial A6 and A7 was taken as a measure of delay cost (*Delay Cost*). The sum of all errors on the recognition portion of the RAVLT (recognition B, SA, PA, SB, and PB) was summed into a single measure of recognition errors (*Recognition Errors*). These five aggregate measures, along with the total score on the *Story Recall* measure, constituted the episodic memory variables.

As mentioned above, reasoning measures administered before BWGU training included the *Visual Puzzles* and *Matrix Reasoning* sub-measures of the Wechsler Adult Intelligence Scale, 4th edition (Drozdick et al., 2012). Participants' total score on each of these respective measures constitutes the reasoning variables in this analysis.

#### Assessment of regional gray matter volumes

Cortical reconstruction and volumetric segmentation of the structural MRI images taken at baseline were conducted with the FreeSurfer 6.0 image analysis suite (Desikan et al., 2006; http://surfer.nmr.mgh.harvard.edu/). FreeSurfer 6.0 was selected over prior versions of FreeSurfer, as that version of the program has been demonstrated to significantly mitigate segmentation errors known to be present in previous versions (Brown et al., 2020; Srinivasan et al., 2020). To further lessen the impact of segmentation errors potentially resulting from Fressurfer's method of automated segmentation, aggregate volumes were used when appropriate, as described below.

Gray matter regions with established links to cognitive control, especially working memory updating and complex skill learning in older adults, were selected as regions of interest to reflect the cognitive demands of the BWGU; these regions included superior, middle, and inferior frontal gyri (Adólfsdóttir et al., 2014; Qin and Basak, 2020), middle temporal gyrus (Zhu et al., 2019), anterior cingulate cortex (Basak et al., 2011; Qin and Basak, 2020), and premotor cortex (Basak et al., 2011). Additionally, the volumes of the hippocampus and striatum were included, due to the known involvement of these regions' in declarative (Burgess et al., 2002; Lim et al., 2020) and procedural learning (Saint-Cyr and Taylor, 1992; Erickson et al., 2010; Doppler et al., 2019; Simonyan, 2019).

FreeSurfer volume outputs corresponding to each of these above regions were summed for each participant to produce an estimated volume of that region for that participant. Striatal volume (*Striatum*) was estimated by summing the separate volume outputs for the caudate, putamen, and nucleus accumbens. Volume estimates of the inferior frontal gyrus (*IFG*) were created by summing the respective volume estimates for the pars opercularis, pars orbitalis, and pars triangularis. The rostral middle frontal and caudal middle frontal volumes estimates of the middle frontal gyrus (MFG) were summed into a single volume estimate of that region. Similarly, rostral anterior cingulate and caudal anterior cingulate volumes output by the program were summed into a single volume estimate of the anterior cingulate cortex (*ACC*). As FreeSurfer does not distinguish between premotor and supplementary motor volumes, the output volume of the precentral gyrus as a whole (*Precentral*) was utilized in this study. The FreeSurfer volume estimates of the superior frontal (*SFG*) and middle temporal gyri (*MTG*), as well as the *Hippocampus*, were used as outputs to represent those regions.

## Results

### BWGU adherence and training outcomes

All participants in the BWGU training arms were instructed to play 20 h of BWGU over the 2-month training period, but self-monitored and self-reported their training time for the duration of the intervention. As a result, a high amount of variance was observed in terms of total training time (*M*_*Time*_ = 17.35 h, *SD*_*Time*_ = 5.93 h). In total, 23 participants successfully reached 20 h of training time with the BWGU paradigm. Of those participants who did not complete the full 20 h of training, five participants explicitly discontinued training (*M*_*Time*_ = 3.48 h, *SD*_*Time*_ = 1.02 h). The remaining nine participants self-reported that they had completed 20 h of training time, but in fact had not when the electronic records of their training time were assessed (*M*_*Time*_ = 16.01 h, *SD*_*Time*_ = 2.95 h).

As stated above, participants were required to complete a daily survey of subjective wellbeing and sleep each time they activated the training program. On average, participants completed 29 surveys over the course of the training period (*M*_*Survey*_ = 29.22, *SD*_*Survey*_ = 14.11), with an average periodicity of one survey every 0.67 h of training (SD_*SurveyTime*_ = 0.32). The number of surveys completed highly correlated with the total training time (*r* = 0.67, *p* < 0.001).

To assess if our variables of interest significantly differed between those participants who completed training and those who did not, we next ran a series of one-way ANOVAs comparing those participants who fully completed the training (*20*+), those who completed the training at under 20 total hours (>*20*), and those who discontinued training (“Discontinued”). Variables assessed in this way included age, *MoCA* score, years of education, *CRIq*, and all of our cognitive variables of interest (*RAVLT* sub-measures, *Matrix Reasoning, Visual Puzzles*, and *Story Memory*). These one-way ANOVAs demonstrated a marginally significant difference between the three completion groups in the *RAVLT Total Learning* and RAVLT *Interference Cost* measures: *TotalLearningF*_(2/34)_ = 2.83, *p* = 0.073; *InterferenceCostF*_(2, 34)_ = 3.06, *p* = 0.06. *Post-hoc* comparisons using Tukey's method demonstrated that, in both cases, these effects were driven by marginal differences between the *discontinued* group and the other groups. The group that discontinued training demonstrated a marginally lower *Total Learning* than both the *20*+ (*p* = 0.93) and >*20* (*p* = 0.76) groups, as well as a marginally higher *Interference Cost* than both the *20*+ (*p* = 0.6*5*) and >*20* (*p* = 0.76) groups. Those that completed training at greater or less than 20 total hours did not differ on these two measures (*Total Learning p* = 0.878; *Interference Cost p* = 0.958), and no other systematic differences in our variables of interest were detected between completion groups.

On average, participants reached level 51 of the BWGU paradigm, the coarsest measure of maximal attainment in this training paradigm, before ceasing training (*M*_*Level*_ = 51, *SD*_*Level*_ = 18.22), with subjects reaching maximal performance at ~11 h of training on average (*M*_*TimeHLR*_ = 11.36, *SD*_*TimeHLR*_ = 5.86). A total of nine participants (24.3% of the sample) reached the maximum difficulty level allowed by the program (72) over the course of the training period. On average, participants completed ~468 individual blocks of the BWGU paradigm throughout the training period (*M*_*Blocks*_ = 467.97, *SD*_*Blocks*_ = 262.23), with each block lasting an average of 2.25 min (*M*_*BlockTime*_ = 2.25, *SD*_*BlockTime*_ = 1.21). Predictably, both highest level reached and number of blocks completed highly correlated with the total training time: *HLR r(37)* = 0.5, *p* = 0.002; *Blocks r(37)* = 0.59, *p* < 0.001.There were no significant differences between the two BGWU arms regarding the total hours played [*t*_(36)_ = 0.2, *p* = 0.84], highest level reached [*t*_(36)_ = 0.79, *p* = 0.43], or number of blocks completed [*t*_(36)_ = −0.29, *p* = 0.77]. A summary of participant training statistics can be found in Table 1.

**Table 1:** Summary statistics for demographic variables, cognitive measures, and the BirdWatch Game—Unity (BWGU) learning measures.

**Measure**	**Mean (SD)**
**Demographics**
Age	71.57 (4.23)
Female	0.54
Education (years)	17.35 (3.15)
MoCA	27.89 (1.56)
**Cognitive measures**
*CRIq*	130.66 (34.66)
*RAVLT Learning Total*	48.51 (12.25)
*RAVLT Interference Cost*	2.12 (1.95)
*RAVLT Delay Cost*	0.27 (1.54)
*RAVLT Recognition Errors*	2.03 (3)
*Matrix Reasoning*	16 (4.06)
*Visual Puzzles*	12.51 (3.88)
*Story Memory*	13.68 (5.52)
**BWGU learning measures**
Time trained (hours)	17.35 (5.93)
Blocks completed	467.5 (262.23)
HLR	51 (18.2)
Overall learning (growth)	639.42 (348.27)
Early learning (growth)	712.67 (401.74)
Middle learning (slope)	3.08 (4.51)
Late learning (slope)	0.71 (2.02)

### Assessment of the relationship between cognitive reserve and cognitive ability prior to BWGU training

To assess if the cognitive reserve was related to baseline cognitive measures, we ran a series of partial correlations between the *CRIq* measure and the pre-training cognitive measurements (*RAVLT: Total Learning, Interference Cost, Delay Cost, Recognition Errors; Matrix Reasoning; Visual Puzzles; Story Memory*), controlling for *Age*. *CRIq* did not demonstrate any significant correlation with *RAVLT* sub-measures, *Total Learning r*(29) = 0.16, *p* = 0.377; *Interference Cost r*(29) = −0.15, *p* = 0.43; *Delay Cost r*(29) = 0.21, *p* =0.267; *Recognition Errors r*(29) = −0.32, *p* = 0.082, nor with *Matrix Reasoning, r*(29) = −0.01, *p* = 0.964, or *Visual Puzzles, r*(29) = 0.04, *p* = 0.827, or *Story Memory, r*(29) = 0.02, *p* = 0.93. These results indicate that cognitive reserve, as measured by the CRIq, is unrelated to pre-training (baseline) cognitive ability in this study.

### Effect of individual differences in baseline cognition and cognitive reserve on BWGU learning

To assess the impact of variance in baseline cognitive measures on learning of the BWGU task, a series of stepwise multiple regressions were conducted with participants' learning variables (*Overall, Early, Middle*, and *Late Learning*) as dependent variables. In each of these regressions, the cognitive predictors (*RAVLT: Learning Total, Interference Cost, Delay Cost*, and *Recognition Errors*; *Story Memory*; *Matrix Reasoning*; and *Visual Puzzles*) were entered in a stepwise fashion until only significant predictors remained.

*Overall Learning* was found to be marginally predicted by a model containing only *Story Memory, R*^2^ = 0.14, *F*_(1, 35)_ = 5.86, *p* = 0.021, *Story Memory* β = 22.34, *t*_(35)_ = 2.21, *p* = 0.034. Similarly, *Early Learning* was found to be significantly predicted by a model containing only *Story Memory, R*^2^ = 0.16, *F*_(1, 35)_ = 6.48, *p* = 0.015, *Story Memory* β = 28.78, *t*_(35)_ = 2.55, *p* = 0.015. Models were not successfully fitted to *Middle* or *Late Learning*, as no combination of the examined predictors produced a model with *p*<*0.1*. To assess if the above relationships co-varied with *Age*, we conducted a series of two-step hierarchical regressions predicting *Overall Learning* and *Early Learning*, respectively. *Age* was entered as a covariate in step 1 of these analyses, with *Story Memory* entered in step 2. In the analysis correcting for age, *Overall Learning* was found to be marginally significantly predicted by a model containing both *Age* and *Story Memory, R*^2^ = 0.16, *F*_(2, 34)_ = 3.26, *p* = 0.051. Within the model, only *Story Memory* was significant, β = 22.34, *t*_(34)_ = 2.21, *p* = 0.034. Similarly, *Early Learning* was found to be significantly predicted by a model containing *Age* and *Story Memory, R*^2^ = 0.31, *F*_(2, 34)_ = 7.72, *p* = 0.002, where both *Age* and *Story Memory* significantly contributed to that model in the expected directions, *Age:* β = −38.23, *t*_(34)_ = −2.78, *p* = 0.009; *Story Memory:* β = 22.37, *t*_(34)_ = 2.22, *p* = 0.033.

Next, a series of regressions were used to assess the influence of cognitive reserve (*CRIq)* on BWGU learning. As with the assessment of cognitive predictors, one regression was performed with *Overall, Early, Middle*, and *Late Learning* as respective dependent variables. In these regressions, *Age* was entered in step 1 as a control variable, followed by *CRIq* in step 2 as the variable of interest. *CRIq* did not significantly predict *Overall Learning, R*^2^ = 0.06, *F*_(1, 30)_ = 1.74, *p* = 0.197, or any of the discrete learning periods examined [*Early Learning: R*^2^ = 0.03, *F*_(1, 25)_ = 1.04, *p* = 0.316; *Middle Learning: R*^2^ = 0.01, *F*_(1, 35)_ = 0.16, *p* = 0.69; *Late Learning: R*^2^ = 0.01, *F*_(1, 25)_ = 0.23, *p* = 0.639]. Note that the combination of between-subject variance in training time and the lack of CRIq data for some participants resulted in these analyses having substantially lower *n* as compared to the analysis of cognitive predictors (overall and early learning: *n* = 37 for cognitive predictors, *n* = 32 for CRIq; middle learning: *n* = 32 for cognitive predictors, *n* = 27 for CRIq; late learning *n* = 31 for cognitive predictors, *n* = 27 for CRIq).

### Effect of individual differences in gray matter volume on BWGU learning

To assess the impact of variance in regional gray matter volumes on learning of the BWGU task, a series of multiple repressions were conducted with participants' learning variables (*Overall, Early, Middle*, and *Late Learning*) as dependent variables. In each of these regressions, the gray matter volumes from the baseline imaging session (left and right *SFG, MFG, IFG, ACC, Precentral, MTG, Hippocampus*, and *Striatum* volume) were entered in a stepwise fashion until only significant predictors remained. These analyses produced a significant model of *Early Learning* (*R*^2^ = 0.16, *F*_(1, 28)_ = 5.36, *p* = 0.028), where the volume of the left *IFG* was the sole contributor (β = 0.109, *t*_(28)_ = 2.31, *p* = 0.028). To evaluate if the relationship between left *IFG* volume and *Early Learning* is significant even after controlling for nuisance variables, a stepwise regression was conducted with *Early Learning* as the dependent variable, Age and estimated total intracranial volume (eTIV) as covariates in step 1, and the left IFG volume in step 2. This resulted in a significant model that predicted *Early Learning* (*R*^2^ = 0.28, *F*_(3, 26)_ = 3.42, *p* = 0.032), with both *Age* and left *IFG* volume as marginally significant predictors, *Age*: β = −32.75, *t*_(27)_ = −1.96, *p* = 0.061; left *IFG*: β = 0.1, *t*_(27)_ = 1.9, *p* = 0.064.

### Combined effects of cognitive and gray matter volume predictors on BWGU learning

The above analyses identified one significant cognitive predictor (*Story Memory*) and one significant brain structure predictor (the volume of the left IFG) of early learning. The influence of *Story Memory* on Early Learning contributed to its influence on Overall Learning. To evaluate the combined effects of these predictors, we conducted two stepwise regressions with *Overall* and *Early Learning* as respective dependent variables. In both regressions, *Age* and *eTIV* were entered in step 1 as control variables, and both *left IFG* and *Story Memory* were entered as variables of interest in step 2. This combinatorial model was found to significantly predict *Early Learning, R*^2^ = 0.44, *F*_(4, 25)_ = 4.82, *p* = 0.005, with both *Story Memory* and *left IFG* contributing significantly to the model, *Story Memory*, β = 35.59, *t*_(25)_ = 2.6, *p* = 0.016; *left IFG*, β = 0.11, *t*_(25)_ = 2.25, *p* = 0.033. This combined model was also found to significantly predict *Overall Learning, R*^2^ = 0.38, *F*_(4, 25)_ = 3.86, *p* = 0.014, with both *Story Memory* and *left IFG* contributing significantly to the model, *Story Memory*, β = 41.73, *t*_(25)_ = 3.44, *p* = 0.002; *left IFG*, β = 0.09, *t*_(25)_ = 2.02, *p* = 0.054.

### Impact of daily context on daily BWGU performance: A time series forecasting analysis

To assess the individual-level influence of daily psychosocial factors on performance-over-time, we ran a series of auto-regressive integrated moving average (ARIMA) analyses using Simple Score as the dependent variable, Training Day as the indexing variable, and Wellness, Stress, Busyness, Mood, and Sleep as independent variables. This analysis was run independently for each participant, allowing for individual assessment of the impact of each moderator on performance-over-time. A total of three participants entered the same response for one or more of the psychosocial context questions for the entire duration of their training, resulting in those psychosocial variables exhibiting zero variance for those participants. Thus, these invariant variables were removed from those participants' models.

These ARIMA analyses were accomplished using the “forecast” package (Hyndman and Khandakar, 2008; Hyndman et al., 2021) for R (R Core Team, 2013). Instead of setting the AR, I, and MA, parameters of the ARIMA models *a piori*, the auto.arima function of the “forecast” package was used to procedurally select the ARIMA model that best fitted each participant's time series. This auto-ARIMA approach examines all possible ARIMA models within the bounds specified, and selects a final model based on the Akaike Information Criterion (AIC), which is a model criterion that accounts for both goodness-of-fit and parsimony of the model (Akaike, 1973, 1987; Sawa, 1978; Bozdogan, 1987, 2000). Maximum parameter bounds for these auto-ARIMA analyses were set to *AR* ≤ 5, *I* ≤ 1, *MA* ≤ 5.

#### Individual ARIMA models of best fit: Prior performance forecasting future performance

The ARIMA models were successfully fit for 34 participants. The ARIMA models did not fit the remaining three participants due to a conjunction of low training time (all three participants discontinued the study prior to completing 5 h of training) and a sparsity of daily survey responses (i.e., longer play sessions resulting in fewer survey prompts occurring during training).

High heterogeneity was observed in the models of best fit across these 34 participants. Ten distinct models were found to be the model-of-best-fit for at least one participant. Of these 10 models, the most common models of best fit were the *AR* = 0, *I* = 0, and *MA* = 0 model (“000”) and the *AR* = 0, *I* = 1, and *MA* = 0 model (“010”), each fitting *n* = 7 participants and together fitting 14 (41%) participants. Both models-of-best-fit feature *AR* and *MA* terms of 0, indicating that the performance of 14 (out of 34) participants on a given day was not strongly influenced by either their prior performances or the moving average of error of their performance on previous days. A summary of all models found to fit at least one participant can be found in Table 2.

**Table 2:** ARIMA models found to significantly explain performance-over-time in at least one participant, grouped by number of occurrences.

**Model**	***AR* term**	***I* term**	***MA* term**	***n***
“000”	0	0	0	7
“100”	1	0	0	5
“200”	2	0	0	2
“300”	3	0	0	1
“010”	0	1	0	7
“110”	1	1	0	4
“210”	2	1	0	2
“011”	0	1	1	2
“111”	1	1	1	2
“211”	2	1	1	1

**Figure 5:**
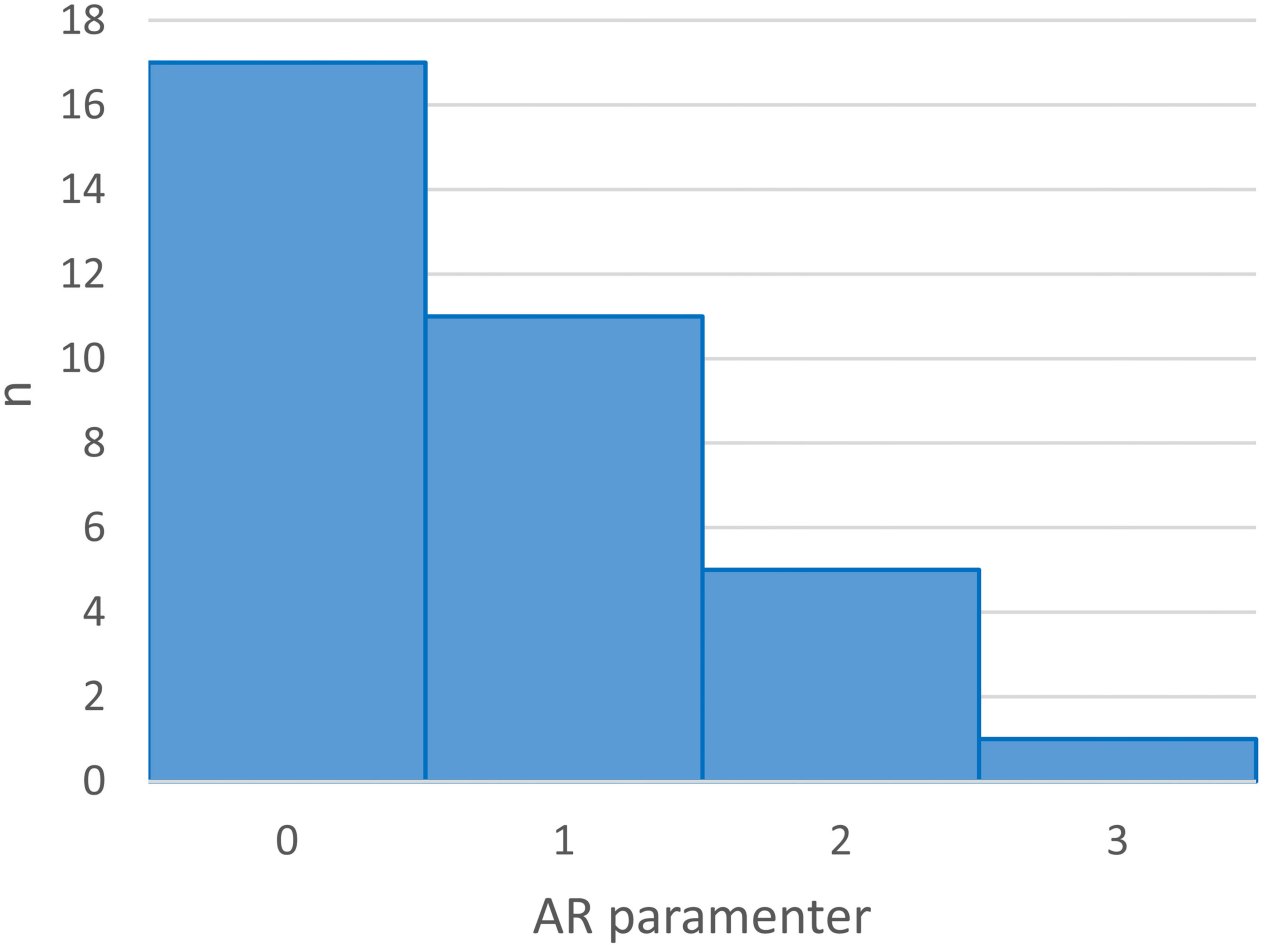
Histogram of AR term values in individual participant's ARIMA model-of-best-fit.

For the remaining participants, 17 (50%) participants' data were best fitted by a model with an *AR* term of one or higher (*M*_*AR*_ = 1.41, range 1–3, see Figure 5), indicating a predictive influence of previous days' performance on the current day's performance. Five participants (14.71%) were fitted by a model counting an *MA* term of 1, indicating that, for those participants, current performance on the BWGU task was predicted by the error term of their previous day's performance. Eighteen participants' data (52.94%) were fit by a model that included an integration (*I*) term of 1, indicating that these participants' performance-over-time exhibited non-stationarity which first-order integration was able to account for (Papoulis, 2002). In total, the performance-over-time of 19 participants (55.88%) was predicted by their previous day's performance, as indicated by a model-of-best-fit which included a non-zero *AR* and/or *MA* term.

#### Individual ARIMA models of best fit: Wellbeing and sleep as predictors of BWGU performance-over-time

As with the model terms of each participant's model-of-best-fit, the value and significance of the psychosocial context moderators and sleep on each participant's performance-over-time also demonstrated notable heterogeneity. Stress significantly predicted performance-over-time at *p* < 0.05, in seven participants (21.21% of the sample), and was found to be the most common single contextual predictor. Wellness significantly predicted performance-over-time on the BWGU task at *p* < 0.05 in four participants (12.12% of the sample). Busyness significantly predicted performance-over-time at *p* < 0.05 in five participants (12.5% of the sample). Mood significantly predicted performance-over-time at *p* < 0.05 in four participants (12.12% of the sample). Sleep also significantly predicted performance-over-time (*p* < 0.05) in three participants (8.82% of sample).

In total, 17 (50%) of the sample demonstrated performance-over-time which was predicted by one or more of the examined psychosocial context variables and sleep, whereas the remaining 17 (50%) participants demonstrated no such relationship.

For participants who exhibited significant relationships between the psychosocial context variables (including sleep) and BWGU performance-over-time, all five variables demonstrated a negative relationship with performance: *Wellness M*_β_ = −200.67, σ_β_ = 192.43; *Stress M*_β_ = −1,012.94, σ_β_ = 1,175.51; *Busyness M*_β_ = −201.64, σβ = 295.99; *Mood M*_β_ = −292.19, σ_β_ = 365.52; *Sleep M*_β_ = −775.64, σ_β_ = 1,063.64. These results demonstrate a highly individualized effect of the examined psychosocial variables on BWGU performance-over-time, including half of our sample for whom performance does not appear to be influenced by the psychosocial context variables examined. Full model reports for each participant can be found in the Supplementary material.

## Discussion

The present study was designed to investigate the cognitive and brain structure correlates of learning a novel gamified computerized working memory task (the BWGU), in order to determine if this game is used as an intervention, what is its potential for far transfer to reasoning and episodic memory and to induce brain plasticity. The results presented above identify one cognitive measure and one structural volume predictor of learning on the BWGU, even after controlling for individual differences in age, specifically of learning within the first 5 hours of the task. In terms of cognitive performance, participant's score on *Story Memory*, a measure of episodic memory, positively related to the participants' learning rates during the first 5 h of practice on the BWGU. In terms of structural volume predictors, estimated gray matter volumes (GMVs) of the participants' left inferior frontal gyrus (IFG) were predictive of learning of the BWGU task during the same period, even after controlling for age.

The *Story Memory* task is a modification of a word-list-recall episodic memory task, with the world list forming a narrative of a coherent episode (Folstein et al., 1975). The strong narrative aspect of the *Story Memory* paradigm may partially explain why performance on that measure was predictive of performance on the BWGU task specifically. One of the modifications made to the BWGU paradigm to increase its efficacy over a traditional n-match was the application of the “bird watching” narrative to the task. It is possible that this narrative operated as a contextual framing device that facilitated performance on the task. If that is the case, the ability to represent and elaborate on this narrative in a way that supports memory, indicated by higher *Story Memory* performance, may have allowed participants to learn the BWGU task at an increased rate.

A past study by Beaunieux et al. (2009) found a somewhat similar pattern of results regarding episodic memory and novel task learning to what was found in the present study. Beaunieux et al. (2009) found that measures of both working and episodic memory predicted a successful acquisition of a novel reasoning task (the Tower of Toronto, Saint-Cyr et al., 1988) over four training sessions. Additionally, Beaunieux et al. (2009) found that episodic memory deficits in older adults (aged 65+ years) in particular, as compared to their younger adult cohort, were negatively predictive of learning on the reasoning task. From this perspective, the results of this study can be interpreted as a specific case of cognitive reserve: degree of retention (i.e., degree of reserve) of episodic memory function, measured in this study by the *Story Memory* measure, may have facilitated learning of the BWGU Task, much as Beaunieux et al. (2009) theorized that preserved episodic memory function did on the Tower of Toronto task in their study.

If the relationship between *Story Memory* and BWGU learning allows for speculation for transfer from training, it is possible that training older adults on BWGU, especially for 5 h or so, may engender transfer to *Story Memory*. This hypothesis is supported by Basak and O'Connell (2016), where 5 h of unpredictable n-match training engendered greater transfer to *Story Memory* recall than the predictable n-match training in older adults. Importantly, faster learning rates were related to greater improvements in *Story Memory*.

Regarding gray matter volume, left inferior frontal gyrus volume significantly predicted learning of the BWGU. As with the *Story Memory* task, left IFG volume was not only found to specifically predict learning during the early phase of the training (hours 1–5), but also significantly contributed to a model predictive of overall learning along with the *Story Memory* measure. Considering the IFG's well-documented role in language processing (Hagoort, 2013; Fedorenko and Thompson-Schill, 2014), the conjunction of left IFG volume and *Story Memory* performance in predicting BWGU task learning strongly suggests that language processing contributes to the learning of the BWGU task. This is a plausible relationship if it is assumed that participants tended to use a verbalization or narrative-based strategy to aid in learning the BWGU task, such as assigning names to the otherwise un-named bird stimuli or applying/embellishing a narrative as a framing device to aid in memory and retrieval of the most recent bird stimuli observed. However, as no strategy self-reports were collected from participants for this study, we cannot assume this is the case. In the absence of confirmation of a language-based strategy for engaging with the BWGU task, exactly how individual differences in language processing would contribute to the learning of the BWGU task remain nebulous.

The inferior frontal gyrus is not, however, exclusively dedicated to language processing: there is ample evidence that it is involved in expressing cognitive control over memory processes more generally. A recent fMRI study by Qin and Basak (2020) found that the IFG is activated not only in younger but also in middle-aged and older adults during the unpredictable two-match task, where digits needed to be retrieved and continuously updated, along with other frontal and parietal regions that are implicated in cognitive control and working memory. Badre and Wagner (2007) concluded based on a review of the literature available at the time that IFG is specifically involved in enforcing cognitive control on the memory retrieval process, a capability essential to the expression of language but not unique to that process (Fedorenko and Thompson-Schill, 2014). A model proposed by Hagoort (2013) specifies that the IFG serves to integrate information from regions of the brain involved in attentional, integrational, and memory processes in a way that allows for precise control of language. This body of work suggests that the IFG is heavily involved in the cognitive control processes of memory retrieval and updating, which are generalizable to language and other tasks. From this perspective, the observed relationship between greater gray matter volume in the left IFG and faster learning of the BWGU task can be interpreted not as dependent on language processing specifically, but that individuals with greater left IFG volume exhibit better cognitive control over their memory retrieval processes during training, thereby producing swifter learning of the task.

Importantly, even when considered together, these predictors (*Story Memory* and Left IFG) were independent contributors to the learning rate across the 20-h training session, even after accounting for age. They were also predictive of learning rates within the first 5 h of training. No significant predictors of the “middle” (hours 5–10) or “late” (hours 10–20) period of training were identified. Model fit and significance were greater when fitting the *Story Memory* + *IFG* model to *Early Learning* compared to *Overall Learning* (Δ*R*^2^ = 0.06), which suggests the pattern seen in overall learning may in fact be driven by the contribution of early learning to that variable. Indeed, a simple linear regression confirms that variation in *Early Learning* significantly explains ~68% of the variance in *Overall Learning* (*R*_2_ = 0.68, *p* < 0.001), with another 17% of the variance being accounted for when *Middle* and *Late Learning* periods are added to the model (*R*_2_ = 0.85, *p* < 0.001). These results would appear to confirm that learning within the first 5 h of training on the BWGU was the primary determinant of overall learning on that task.

The above relationship confirmed, why then were the observed structural and cognitive predictors of learning of the BWGU not related to learning rates in hours 5–10 or 10–20 of the training? The learning model proposed by Ackerman (1988) states that the first phase of learning is primarily determined by cognitive factors, with later learning primarily determined by the development of strategy and automatization of task-relevant responses. Considering that the potential predictors of learning that were examined in this study consisted of (a) cognitive predictors and (b) gray matter volume of regions related to the training task and cognitive predictors, it is no surprise then that any relationship uncovered would pertain to the early learning period specifically. The present study did not assess strategy formation or use by participants, and as such does not include a variable with sensitivity to Ackerman's strategy-dependent second phase of learning. The automatization-dependent third stage of Ackerman's model predicts stability of performance but improvement of response time on time-sensitive tasks. This flattening of performance is likely captured in the “late” learning period of the present study, defined by asymptotic performance on the BWGU tasks, but again no time-based variables sensitive to the development of automatized processing were examined in the analysis presented here. In short, strategy-based learning and automatization may well have been facilitated over 20 h of training on the BWGU task, but the game score analyzed here was not sensitive to those processes. This is not to say that this study's findings related to early learning are spurious. Rather, it should be recognized that variance in individual learning rates from strategy-based or automatic processes, both of which hypothetically contribute to later learning, are likely not accounted for in these analyses due to predictor and outcome variables utilized in this study.

Ackerman (1988) model of procedural learning offers an explanation as to why cognitive predictors of early learning were found in this study generally, but not why episodic memory measure and left IFG volume specifically predicted early learning of the BWGU task. Taken together, these predictors appear to reflect participants' ability to apply cognitive control to memory retrieval and, as needed, update the memory to encode it even for information that is tracked over a few seconds. As discussed above, aspects of the BWGU task itself, such as heavy emphasis on working memory updating, incorporating narrative framing device, as well as the known sensitivity of the *Story Memory* measure to n-back-based training (Basak and O'Connell, 2016) may well account for this. However, past work by Beaunieux et al. (2006) identified both episodic memory and cognitive control as indicative of learning a reasoning task (the Tower of Toronto). Beaunieux et al. (2006) concluded that episodic memory and executive function contributed to the first stage of learning in Ackerman's model. While the authors do not fully support that position based on the evidence provided by Beaunieux et al. (2006) that a similar pattern of predictors was found to relate to early learning on both the Tower of Toronto and the BWGU task suggests that these results might be generalizable beyond these select tasks, which is certainly worthy of future study. This study showed that in older adults who trained on a novel gamified, individualized-adaptive working memory updating intervention, the BirdWatch Game—Unity, for about 20 h, individual differences in a measure of episodic memory and the volume of left inferior frontal gyrus predicted individual's learning rate. These relationships were specifically applicable to the early phase of novel game learning, where individuals display rapid gains in game performance.

Importantly, neuro-cognitive predictors of skill learning on a task, such as BWGU, can inform us about the potential transfer mechanisms that can result from training on such skills. Another significance of this study is the potential identification of individuals who may benefit most from BWGU training.

Notably, the included measure of cognitive reserve (CRIq) did not reliably predict overall learning of the BWGU task, nor learning in any of the discrete training periods examined. This is perhaps not surprising as the cognitive reserve is typically conceptualized as a protective factor (Tucker and Stern, 2011; Opdebeeck et al., 2016), rather than a factor that facilitates cognitive function, and the existing evidence linking cognitive reserve to task learning is somewhat weak (Lojo-Seoane et al., 2020). This is not to say that the study has definitively produced no evidence of reserve contribution to the learning of the BWGU task: as mentioned earlier, the observed relationship between *Story Memory* recall and BWGU learning may well be evidence of cognitive reserve, especially considering the degree of decline in episodic memory typically observed in older adults (Park et al., 2002; Rozas et al., 2008). A similar argument can be made regarding brain reserve. However, in the absence of cognitive or brain structure measurements taken from these participants earlier in life, these reserve arguments cannot be directly supported. Importantly, cognitive reserve is typically indexed by measures of life-time cognitive activity and educational attainment, and has been found to interact with cognitive training-related gains in cognition in healthy aging (for a meta-analysis, see Basak et al., 2020). It can be concluded, however, that cognitive reserve as measured by the CRIq as a sum of educational attainments and self-report aggregate of life experience does not relate to learning of the BWGU task.

The second goal of this study was to determine whether fluctuating psychosocial context variables and sleep duration influenced performance-over-time on the BWGU task. The most general hypothesis that sleep and the psychosocial variables examined would influence performance-over-time was demonstrably true for 50% of the sample, or 17 total participants, while the other 50% of the sample demonstrated no such relation. This, obviously, limits the conclusions we can draw based on this evidence. We cannot declare that a random participant from this sample would be more likely than not to be affected by one or more of the examined psychosocial context variables, due to simple probability. However, this result still allows for some definite conclusions to be drawn.

First, that performance-over-time of 50% of the sample of this study was influenced by at least one of the daily survey measures (that is, sleep, stress, busyness, mood, or wellbeing) is far from a negligible fraction. Indeed, if we assume that these results are generalizable, then it is fairly likely that performance-over time on the BWGU task will be influenced by one or more of these factors for a given participant. Additionally, there are likely undetected moderators which partially determine whether a given participant's performance is influenced by a given psychosocial context variable or sleep, which are important to further investigate considering how pervasive the influence of these psychosocial context variables and sleep are on cognition. Considering the well-documented negative impact of disrupted sleep (Holanda Júnior and de Almondes, 2016; Lo et al., 2016; Rana et al., 2018; Zavecz et al., 2020) and high stress (Shields et al., 2016; Plieger and Reuter, 2020) on cognitively demanding tasks in the real world, understanding what variables may moderate this relationship is of substantial real-world importance. The results of the present study indicate that sleep and the psychosocial context variables examined in this study can have an impact on the performance and learning of complex tasks, which is warranted enough for further investigation.

Second, while the generalization of these results is problematic, these models do offer significant explanatory power with regard to each individual participant. This has potential utility within the cognitive training domain as a method of assessing the individual needs of a participant, and providing cognitive training that is individually adaptive to those needs. Accurate models were fitted for participants who completed as little as 3 h of training, and for all participants who completed more than 5 h of training. Within the timescale of a long-term cognitive intervention, which typically involves 10-20 h of training (Basak et al., 2020), an analysis like the one performed in this study could be conducted with sufficient remaining time to provide individuated participant feedback or adjust the prescribed training, to account for any significant psychosocial effects observed. This is an alternate approach to individualized-adaptive training to the closed-loop strategy implemented in the design of the BWGU paradigm, where training difficultly was manipulated with respect to performance metrics (block-wise d' and consecutive failures), but not daily sleep or perceived wellbeing. Our current approach is agnostic to idiosyncratic influences on individual subjects, under the assumption that such sporadic daily influences are reflected in each participant's overall performance. Identifying and accounting for specific factors influencing performance-over-time, which the method of analysis presented in this study could facilitate, may serve as an effective additional method of adaptive training independent of the performance-focused method implemented in BWGU. Importantly, findings from the time series forecasting analysis provide evidence for why the individualized-adaptive approach to training has been generally successful at inducing positive training outcomes (Payne et al., 2011; Brehmer et al., 2012; Cuenen et al., 2016). A wide array of patterns of psychosocial influence were observed even within the age and geographically restricted sample utilized in this study, and it can be assumed with some confidence that individuals undergoing any form of cognitive training or intervention are subject to a similarly wide array of moderating influences.

The analysis presented in this study also demonstrated that 50% of the sample (*n* = 17) exhibited performance-over-time that was reliably predictable by previous performance, either through direct auto-regression of past performance onto a given day's BWGU score, or *via* a moving average of error terms. The finding that for 50% of our sample, current BGWU performance was not reliably predictable from past performance is interesting, as it suggests that other factors are primarily responsible for performance-over-time in this large proportion of the sample. As discussed, psychosocial context variables demonstrably accounted for variance in performance in half of our sample, which includes 11 of the 17 participants for whom past performance did not relate to performance-over-time. However, this still leaves six participants for whom none of the examined variables, including their own performance, was predictive of variability in performance-over-time. The only conclusions that can be drawn about what these other factors might be are that they (a) have periodicity longer than the training period observed or (b) are transient events, as otherwise evidence of any such predictable influence would be detectable in the auto-regressive or moving average analysis. In light of these findings, it is clear that individual influences on performance-over-time on a complex task like the BWGU task are highly varied, and that they can be very influential. Further investigation of how these individual-level factors can be identified, modeled, and accounted for can only be a boon to efforts to develop efficacious, individualized cognitive interventions.

As already mentioned, the design of this study limits some of the conclusions we are able to draw from these results, and these design limitations can be improved upon in future iterations of this work. First, the present study did not take participant strategy into account. This is a particularly pertinent limitation to the findings of this study considering (a) the possibility that participants were utilizing a verbalization or narrative-based strategy to aid learning of the BWGU task, and (b) the theoretical relevance of strategy generation toward procedural task learning. A *post-hoc* self-report could potentially allow for insight into the effect of strategy on BWGU learning; however, this self-reported approach would need a much larger sample size of 250 or more given the variability of self-generated strategy reports and associated variables of interest, such as personality factors (e.g., openness to experience), cognitive flexibility, IQ, etc. Such a research agenda is challenging to implement in cognitive interventions that last for months and include brain measures. Another approach to studying the role of strategy could be a strategy manipulation applied *via* varied participant instructions, although this would require an in-lab intervention and a much larger multi-arm RCT that would have similar limitations of the feasibility of study implementation in terms of time and resource as described before. Second, the design of the present study did not allow for a detailed investigation of the influence of cognitive/brain reserve on learning of this task, beyond the retroactive self-report measure utilized by the CRIq. Addressing this shortcoming is somewhat difficult: A longitudinal approach by which trajectories of cognitive/neurological change over time could be calculated before the training period began could potentially enlighten and specify the reserve-learning relationship, but this would require a major expenditure of time and resources to accomplish.

## Conclusion

This study showed that in older adults who trained on a novel gamified, individualized-adaptive working memory updating intervention, the BirdWatch Game—Unity, for about 20 h, individual differences in a measure of episodic memory and the volume of left inferior frontal gyrus predicted individual's learning rate. These relationships were specifically applicable to the early phase of novel game learning, where individuals display rapid gains in game performance. These predictors appear to reflect participants' ability to apply cognitive control to episodic memory functions, especially memory retrieval and subsequently updating the memory to encode it even for information that is tracked over a few seconds as in BWGU. Importantly, neuro-cognitive predictors of skill learning on a task, such as BWGU, can inform us about the potential transfer mechanisms that can result from training on such skills. In fact, prior research in older adults has shown that just 5 h of training on working memory updating, where stimulus sequence appeared in unpredictable order, results in far transfer to *Story Memory* recall, the measure of episodic memory that was found to be a significant predictor in the current study. Taken together, these results suggest that neuro-cognitive predictors of task learning can be informative about whether we can see potential transfer to tasks that have the same neuro-cognitive underpinnings. Another significance of the current study is the potential identification of individuals who may benefit most from BWGU training. Episodic memory is considered to be an early marker of mild cognitive impairment; therefore, it is possible that BWGU training may be beneficial to not only healthy older adults but to build a reserve in a key cognitive function known to be impacted in at-risk older adults, such as those with mild cognitive impairment. Finally, forecasting analysis on the time series of the game shows that day-to-day psychosocial wellbeing and hours of sleep can impact the game performance of that day or of the next day, but only in about 50% of participants in this study. Others did not exhibit any relationship between these daily measures (sleep and wellbeing) and game performance. Large-scale studies are warranted to understand why some older adults show such dependencies, and whether resistance to such dependencies results in the long-term maintenance of cognition. Importantly, data from these time series forecasting suggest that for a large proportion of individuals, the efficacy of the intervention can be improved at an individual level by incorporating sleep and psychosocial factors into the closed-loop individualized-adaptive feedback design. Identification through such modeling of how these individual-level daily variables (task performance, sleep, mood, etc.) impact our learning during an intervention can help us develop more efficacious, individualized cognitive interventions.

## Data availability statement

The datasets presented in this study can be found in online repositories. The names of the repository/repositories and accession number(s) can be found at: https://utdallas.box.com/s/c50xa6jg7u28kmyume070s4jitreecsu.

## Ethics statement

The studies involving human participants were reviewed and approved by University of Texas at Dallas Institutional Review Board. The participants provided their written informed consent to participate in this study.

## Author contributions

CB designed the original BirdWatch Game in Matlab and the cognitive intervention. The game was further developed by CB, ES, and PF as BWGU in the Unity platform. CB developed the study as a Principal Investigator, with the assistance of PF and DP, who were co-investigators on the project, evaluated individual subjects learning rates across various functions, conducted analyses of BWGU arm differences, and edited and co-wrote the manuscript. ES, PS, AS, and SQ collected the data for the clinical trial. PS and SQ performed the MRI preprocessing and FreeSurfer analysis of the structural data under CB's direction. ES performed all other analyses and wrote the first version of the manuscript. All authors contributed to the article and approved the submitted version.

## Funding

This research was supported by a grant from the National Institute on Aging to CB (titled Strategic Training to Optimize Neurocognitive Functions in Older Adults under Award R56AG060052, PI: CB).

## Conflict of interest

The authors declare that the research was conducted in the absence of any commercial or financial relationships that could be construed as a potential conflict of interest.

## Publisher's note

All claims expressed in this article are solely those of the authors and do not necessarily represent those of their affiliated organizations, or those of the publisher, the editors and the reviewers. Any product that may be evaluated in this article, or claim that may be made by its manufacturer, is not guaranteed or endorsed by the publisher.
