# Supplementary material for: Cognitive and structural predictors of novel task learning, and contextual predictors of time series of daily task performance during the learning period
Source: Front Aging Neurosci. 2022 Sep 23;14:936528. doi: 10.3389/fnagi.2022.936528 (PMC9540228; doi:10.3389/fnagi.2022.936528)
Supplement: Supplementary file 1 [file Data_Sheet_1.pdf]

Cognitive and Structural Predictors of Novel Task Learning, and Contextual Predictors of Time-Series of Daily Task Performance During the Learning Period.

Supplementary Material

1 Factor Analysis of Cognitive Variables at Baseline

In order to more fully examine the preexisting relationships between the baseline cognitive variables (including cognitive reserve) examined in this study, we next performed an exploratory factor analysis of the said variables (*CRIq, RAVLT* submeasures*, Matrix Reasoning, Visual Puzzles,* and *Story Memory*) using Varimax rotation (Browne, 2001; Kaiser, 1958). The principal axis factoring method was utilized to extract factors (de Winter & Dodou, 2012; Fabrigar et al., 1999). As the measures examined in this factor analysis theoretically constitute three separable constructs (episodic memory, reasoning, and cognitive reserve), the number of factors for this analysis was set at 3. This number was verified via scree analysis, which demonstrated three factors demonstrating an eigenvalue > 1 (see Figure S1) This factor analysis was facilitated by the "psych" package (Revelle, 2021) for R (R Core Team, 2013).

This three-factor model was found to cumulatively explain 59% of the sample variance, with an overall model fit of .73, and offset (diagonal) model fit of .96. Loadings of each cognitive predictor variable on each of these three components can be found in Table S1. Factor 1 was primarily determined by the *Visual Puzzles* (coefficient = 1.29) and *Matrix Reasoning* (coefficient = .49) measures, corresponding to ***a priori*** reasoning construct. For Factor 2, the RAVLT submeasures, *Learning Total* (coefficient = .83) and *Recognition Errors* (coefficient = -.58), as well as the *Story Memory* measure (coefficient = .62) corresponded to ***a priori*** Episodic Memory construct. Factor 3 was primarily determined by the *Matrix Reasoning* measure (coefficient = .59) and the RAVLT submeasures, *Interference Cost* (coefficient = .57) and *Delay Cost* (coefficient = -.48); this factor does not correspond to any ***a priori*** construct. Importantly, CRIq was not a major contributor to any of these factors, maximally reaching a contribution of .2 to Factor 2 and -.2 to Factor 3.

2 References

Browne, M. W. (2001). An Overview of Analytic Rotation in Exploratory Factor Analysis. *Multivariate Behavioral Research*, *36*(1), 111–150. https://doi.org/10.1207/S15327906MBR3601_05

de Winter, J. C. F., & Dodou, D. (2012). Factor recovery by principal axis factoring and maximum likelihood factor analysis as a function of factor pattern and sample size. *Journal of Applied Statistics*, *39*(4), 695–710. https://doi.org/10.1080/02664763.2011.610445

Fabrigar, L. R., Wegener, D. T., MacCallum, R. C., & Strahan, E. J. (1999). Evaluating the use of exploratory factor analysis in psychological research. *Psychological Methods*, *4*(3), 272–299. https://doi.org/10.1037/1082-989X.4.3.272

Kaiser, H. F. (1958). The varimax criterion for analytic rotation in factor analysis. *Psychometrika*, *23*(3), 187–200. https://doi.org/10.1007/BF02289233

R Core Team (2013). R: A language and environment for statistical computing. R Foundation for Statistical Computing, Vienna, Austria. http://www.R-project.org/.

Revelle, W. (2021). *psych: Procedures for Psychological, Psychometric, and Personality Research.* Northwestern University, Evanston, Illinois. R package version 2.1.6, https://CRAN.R-project.org/package=psych.

Table S1. Results of Exploratory Factor Analysis examining baseline (pre-training) Episodic Memory, Reasoning, and Cognitive Reserve measures.

	Factor 1	*Factor 2*	*Factor 3*
CRIq	.01	0.2	-.21
RAVLT Learning Total	.11	**.83**	.03
RAVLT Interference Cost	-.08	-.3	**.57**
RAVLT Delay Cost	.03	.05	**-.48**
RAVLT Recognition Errors	.06	**-.58**	.32
Matrix Reasoning	**.49**	.22	**.59**
Visual Puzzles	**1.29**	.08	-.08
Story Memory	.09	**.62**	-.07
Factor Eigenvalue	2.33	1.82	1.1
Variance Explained	0.24	0.2	0.15

Supplementary Figure 1. Scree plot of factors present in pre-training cognitive data. Plot based on uncorrected correlation matrix.
