# Supplementary figures and images for: Cognitive and structural predictors of novel task learning, and contextual predictors of time series of daily task performance during the learning period
Source: Front Aging Neurosci. 2022 Sep 23;14:936528. doi: 10.3389/fnagi.2022.936528 (PMC9540228; doi:10.3389/fnagi.2022.936528)

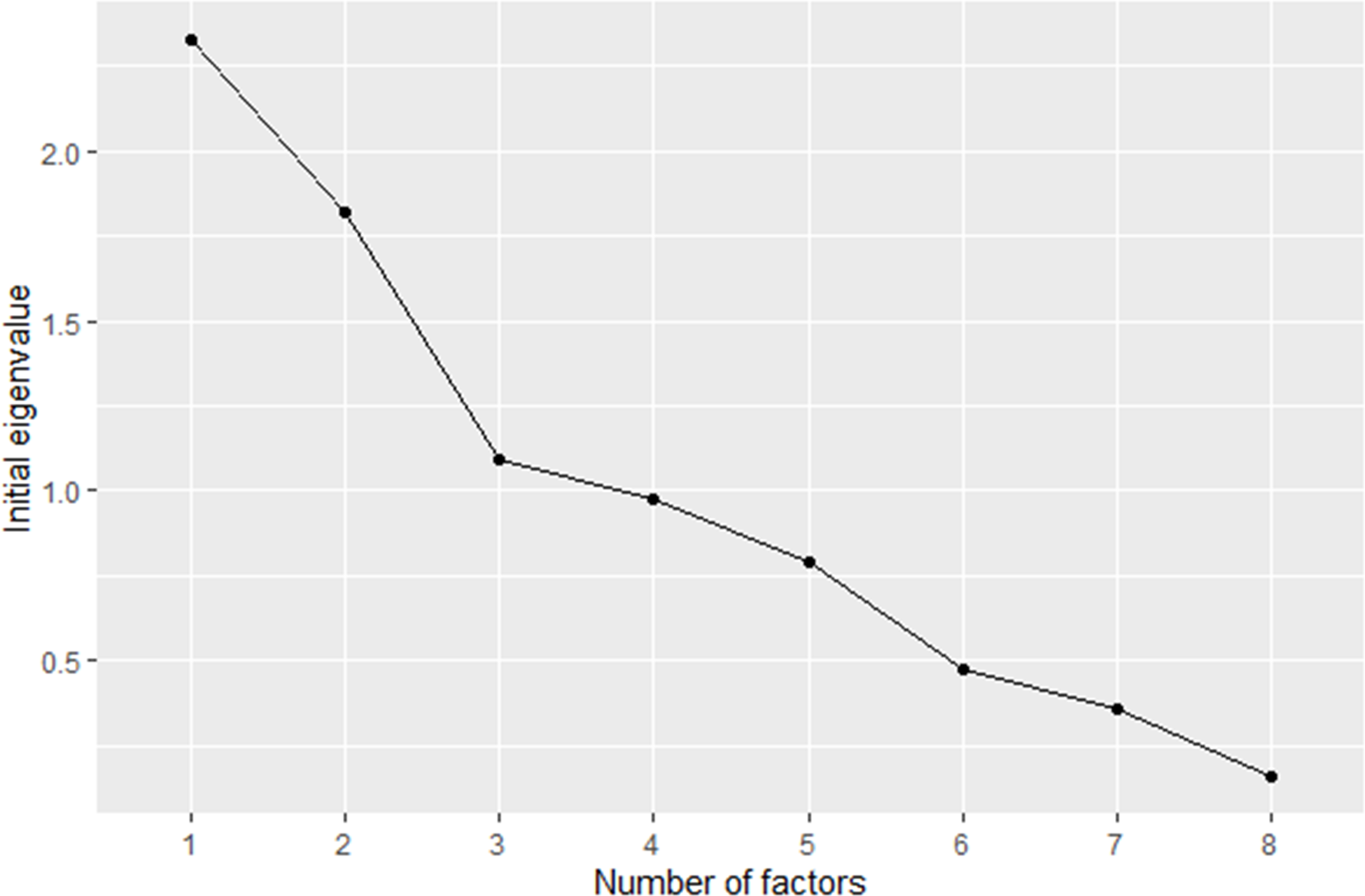

Supplement: Supplementary file 2 [file Image_1.TIF]
